# Supplementary material for: Elucidating variations in outcomes among older end-stage renal disease patients on hemodialysis in Fukuoka Prefecture, Japan
Source: PLoS One. 2021 May 25;16(5):e0252196. doi: 10.1371/journal.pone.0252196 (PMC8148375; doi:10.1371/journal.pone.0252196)
Supplement: Attachment — Submitted filename: Response to reviewer 27032021.docx [file pone.0252196.s002.docx]

Dr. Gregory Tiao

Academic Editor

*PLOS ONE*

27 March 2021

Dear Dr. Tiao,

Thank you for allowing us to submit a revised draft of our manuscript entitled “*Elucidating variations in outcomes among older end-stage renal disease patients on hemodialysis in Fukuoka prefecture, Japan.*”

We appreciate the time and effort that you and the reviewers have dedicated to providing valuable feedback for our manuscript. We are also grateful to the reviewers for their insightful comments. We have made the necessary changes to the manuscript to reflect most of the suggestions and comments. We have also highlighted the changes within the manuscript.

Below, please find our point-by-point responses to the reviewers’ comments and concerns.

We look forward to hearing from you regarding our submission and to respond to any further questions and comments you may have.

Kind regards,

Aziz Jamal

Department of Health Care Administration & Management

Graduate School of Medical Sciences, Kyushu University

Fukuoka Japan.

Email: [azizabduls.jamal@gmail.com](mailto:azizabduls.jamal@gmail.com)

Reviewers' comments

**Reviewer #2**

Thank you authors for addressing the comments raised. Here are the few comments to address:

1. Keywords: Retrospective is not a key word, remove. May you add the word outcome as a key word.

**Author response:**

*Thank you for the suggestion. The key word ‘retrospective’ has been removed and replaced with ‘outcome’.*

2. Methods section in the manuscript: Organization of the methods section is paramount for the readers to follow and understand clearly. May you please reorganize this section in the following order: study design, study location, population (Patient identification), Inclusion and exclusion criteria, sample size, data source, definition of variables, definition of outcomes, data analyses and ethical considerations.

**Author response:**

*Thank you for your kind guidance. We have organized the methods section accordingly. We have ordered the sub-sections as followings: Study design, Study location, Population (Patient identification), Inclusion and exclusion criteria, Sample size, Data source, Definition of variables, Definition of outcomes, Data analyses, Ethical consideration.*

3. Study design and patient identification should be separated.

**Author response:**

*Thank you for your suggestion. We have separated the content accordingly. In the revised manuscript, the explanation of study design and patient identification is provided under two separate subheadings (i.e., Study design, Population (Patient identification)).*

**Reviewer #3**

1. The authors have responded to my suggestions and addressed my concerns. I only have one additional suggestion. The focus of the study is on variation in care. While the authors now dedicate part of the discussion to address the clinical significance of findings about variation, much of the discussion section focuses on crude rates of various health outcomes. Less of a focus on findings from crude rates in the discussion would help to align the discussion text with the manuscripts focus, which is on geographic variation.

**Author response:**

*We appreciate your suggestion. The paragraph has been revised accordingly. We removed the sentences mentioning the crude rates and replaced them with sentences highlighting outcome variations that were observed in several areas (i.e., Kurume, Ariake, Iizuka and Kitakyushu). These observed variations were based on the result of multivariable logistic regression analyses.*

(see Discussion Section, p.25, Line 528—541 )
